# Crosstalk Between MAPK/ERK and PI3K/AKT Signal Pathways During Brain Ischemia/Reperfusion

**DOI:** 10.1177/1759091415602463

**Published:** 2015-10-06

**Authors:** Jing Zhou, Ting Du, Baoman Li, Yan Rong, Alexei Verkhratsky, Liang Peng

**Affiliations:** 1 Laboratory of Metabolic Brain Diseases, Institute of Metabolic Disease Research and Drug Development, China Medical University, Shenyang, P. R. China; 2 Faculty of Life Science, The University of Manchester, UK; 3 Achucarro Center for Neuroscience, IKERBASQUE, Basque Foundation for Science, Bilbao, Spain; 4 University of Nizhny Novgorod, Russia

**Keywords:** brain ischemia, EGF receptor, AKT, ERK, Raf-1, PTEN

## Abstract

The epidermal growth factor receptor (EGFR) is linked to the phosphatidylinositol 3-kinase (PI3K)/protein kinase B (AKT) and Raf/mitogen-activated protein kinase (MAPK)/extracellular signal-regulated kinase (ERK_1/2_) signaling pathways. During brain ischemia/reperfusion, EGFR could be transactivated, which stimulates these intracellular signaling cascades that either protect cells or potentiate cell injury. In the present study, we investigated the activation of EGFR, PI3K/AKT, and Raf/MAPK/ERK_1/2_ during ischemia or reperfusion of the brain using the middle cerebral artery occlusion model. We found that EGFR was phosphorylated and transactivated during both ischemia and reperfusion periods. During ischemia, the activity of PI3K/AKT pathway was significantly increased, as judged from the strong phosphorylation of AKT; this activation was suppressed by the inhibitors of EGFR and Zn-dependent metalloproteinase. Ischemia, however, did not induce ERK_1/2_ phosphorylation, which was dependent on reperfusion. Coimmunoprecipitation of Son of sevenless 1 (SOS1) with EGFR showed increased association between the receptor and SOS1 in ischemia, indicating the inhibitory node downstream of SOS1. The inhibitory phosphorylation site of Raf-1 at Ser259, but not its stimulatory phosphorylation site at Ser338, was phosphorylated during ischemia. Furthermore, ischemia prompted the interaction between Raf-1 and AKT, while both the inhibitors of PI3K and AKT not only abolished AKT phosphorylation but also restored ERK_1/2_ phosphorylation. All these findings suggest that Raf/MAPK/ERK_1/2_ signal pathway is inhibited by AKT via direct phosphorylation and inhibition at Raf-1 node during ischemia. During reperfusion, we observed a significant increase of ERK_1/2_ phosphorylation but no change in AKT phosphorylation. Inhibitors of reactive oxygen species and phosphatase and tensin homolog restored AKT phosphorylation but abolished ERK_1/2_ phosphorylation, suggesting that the reactive oxygen species-dependent increase in phosphatase and tensin homolog activity in reperfusion period relieves ERK_1/2_ from inhibition of AKT.

## Introduction

Receptors to growth factors are functionally linked to mitogen-activated protein kinase (MAPK)/extracellular signal-regulated kinase (ERK_1/2_) and phosphatidylinositol 3-kinase (PI3K)/protein kinase B (AKT) intracellular signaling pathways. When members of epidermal growth factor (EGF) family bind to EGF receptor (EGFR), the EGFR becomes phosphorylated (activated) on tyrosine residues. Subsequently, the guanine nucleotide exchange factor Son of sevenless 1 (SOS1) becomes activated, thus leading to stimulation of the small G-protein Ras. The latter actuates serine/threonine-kinase Raf, the first kinase of the three-tiered MAPK/ERK_1/2_ pathway. There are three Raf isoforms, Raf-1/c-Raf, B-Raf, and A-Raf (for review, see Matallanas et al., 2011), of which Raf-1 has been extensively studied. Raf-1 has several phosphorylation sites, among which phosphorylation of Ser338 is required for Raf-1 activation (Mercer and Pritchard, 2003). Phosphorylation of Ser259, to the contrary, inhibits the MAPK/ERK_1/2_ pathway (Abraham et al., 2000; Yip-Schneider et al., 2000; Dhillon et al., 2002; Adler et al., 2008). PI3K is a lipid protein kinase functionally positioned downstream of Ras. Once activated, PI3K converts membrane-bound phosphatidylinositol (4,5)-bisphosphate into phosphatidylinositol (3,4,5)-trisphosphate. This latter anchors AKT to the plasma membrane and allows its phosphorylation.

In addition to direct stimulation by EGF family members, the EGFR transactivation is operative under various conditions. In the process of transactivation, an increase in intracellular Ca^2+^ concentration ([Ca^2+^]_i_) (Zwick et al., 1997; Piiper et al., 2003; Peng et al., 2012) mediated by activation of G_i/o_ or G_q_ protein-coupled receptors (Pierce et al., 2001; Peng et al., 2003) or by other stimuli leads to Zn^2+^-dependent metalloproteinase-catalyzed shedding of a growth factor of the EGFR family (Cai et al., 2011). This agonist stimulates EGFRs on the same cell or its neighbor(s) and activates two major intracellular signaling cascades represented by the MAPK/ERK_1/2_ and PI3K/AKT pathways (Gu et al., 2007). Cardioprotection of EGFR transactivation has been extensively studied in ischemic preconditioning by exposing the heart to brief cycles of ischemia and reperfusion (for review, see Downey et al., 2007, 2008). EGFR is also involved in reperfusion-induced arrhythmias (Feng et al., 2012). In the brain *in vivo*, hypoxia caused nitric oxide (NO)-mediated increase of EGFR phosphorylation and its activity in cerebral cortex of newborn piglets (Mishra et al., 2010). In middle cerebral artery occlusion (MCAO) model, EGFR phosphorylation was found in the cortex boundary zone at 3, 7, and 14 days after 1-hr transient ischemia (Yang et al., 2011). Inhibition of EGFR with the neutralizing antibody C225 reduced infarct volume and attenuated reactive astrogliosis in MCAO model (Yang et al., 2011). The PI3K/AKT pathway is believed to mediate cell protection (Murphy and Steenbergen, 2008), whereas the functions of MAPK/ERK_1/2_ pathway are more complex. Transient, moderate ERK phosphorylation mediates cell protection, for example, during ischemic precondition (Murphy and Steenbergen, 2008), whereas sustained, extensive ERK phosphorylation may induce cell injury (Martin and Pognonec, 2010). Cell damage in ischemia occurs because of ATP depletion that leads to a loss of cellular ionic homeostasis and membrane depolarization, which, in turn, increases intracellular Ca^2+^ concentration and mitochondrial depolarization, decreases intracellular pH, and triggers massive release of glutamate. In clinical settings, only a small part of patients experience complete reperfusion, with majority having either partial reperfusion or no reperfusion at all (see Zhao et al., 2007, and references thereby). During reperfusion, cells experience extensive oxidative damage because restoration of oxygen results in generation of large amount of reactive oxygen species (ROS; Murphy and Steenbergen, 2008). Further understanding of signaling pathways during ischemia/reperfusion could provide detailed information about mechanisms underlying cell protection or cell injury.

Conceptually, the PI3K/AKT and Raf/MAPK/ERK_1/2_ represent two parallel signal pathways. Interactions between PI3K/AKT and Raf/MAPK/ERK_1/2_ pathways may occur at several different stages and could be either positive or negative. In preliminary experiments, we found that ERK activity was dependent on reperfusion. We hypothesized that the PI3K/AKT signal pathway is involved in the regulation of ERK activity during ischemia or reperfusion. Therefore, in the following study, we investigated the activation of EGFR and its two intracellular signal pathways, MAPK/ERK_1/2_ and PI3K/AKT during ischemia or reperfusion in MCAO model.

## Methods and Materials

### Animals

Adult male Sprague-Dawley rats, weighing 280 to 400 g, were housed in cages on a 12-hr light/dark cycle in a temperature-controlled (23℃–25℃) colony room with free access to food and water. All experiments were carried out in accordance with the USA National Institute of Health Guide for the Care and Use of Laboratory Animals, and all experimental protocols were approved by the Institutional Animal Care and Use Committee of China Medical University.

### Middle Cerebral Artery Occlusion

Focal brain ischemia was induced unilaterally by occlusion of the right middle cerebral artery (MCAO) as previously described (Longa et al., 1989). Briefly, rats were anesthetized with 1% pentobarbital sodium (40 mg/kg, i.p.), and the right common and external carotid arteries were exposed. A 30-mm segment of 4-0 ethilon monofilament (Ethilon; Johnson & Johnson, Woluwe, Belgium) was gently inserted from the external carotid artery into the internal carotid artery lumen and advanced toward the origin of the middle cerebral artery until a slight resistance was felt. The filament had been coated with polylysine, and its tip rounded by heating. The rats were killed after 2 hr of MCAO with/without 0.5, 1, or 2 hr of reperfusion.

Intraventricular injection was performed according to the rat brain atlas by Paxinos and Watson (2007). Rats were anesthetized with 1% pentobarbital sodium (40 mg/kg, i.p.) and were placed in the stereotaxic frame. Midline incision was made to see the bregma suture. The surgical site was cleaned, and the holes were made by an electric dental drill at the right ventricle at 1.0 mm posterior to bregma, 1.5 mm lateral from midline. Stainless steel microinjector was stereotaxically implanted into the hole at 4.0 mm vertically from the skull surface. The microinjector was left in place for 10 min to minimize backflow and then removed. The drugs were gently injected intraventricularly, dissolved in 4 or 5 μl physiological saline, at the following concentrations: 2-(4-morpholinyl)-8-phenyl-1(4H)-benzopyran-4-one hydrochloride (LY294002), 2 mM; tyrphostin 4-(3-chloroanilino)-6,7-dimethoxyquinazoline (AG1478), 100 μM; catalase, 20,000 U/ml; rapamycin, 100 μM; 1,4-diamino-2,3-dicyano-1,4-bis[2-aminophenylthio] butadiene (U0126), 1 mM; triciribine hydrate (TCN), 1 mM; and bisperoxovanadium (BPV), 10 μM. The drugs were injected into the right lateral ventricle 15 min before MCAO or 15 min before reperfusion. With a brain weight in the rat of 1 to 2 g, the intracerebral concentrations are probably at least 100 times smaller.

### Brain Section

At the end of experiment, each brain was quickly removed and collected. Brain sectioning was accomplished according to the protocol previously reported by Ashwal et al. (1998). Briefly, the brains were sectioned into three parts, the first part begun in 3 mm from the anterior tip of the frontal lobe. The second part (4 mm thick) was used for the measurement. Regions from the right and left hemispheres of the second part that corresponded to the ischemic core and penumbra were dissected. A longitudinal cut (from top to bottom) was made approximately 2 mm from the midline through each hemisphere. Then, a transverse diagonal cut was made at approximately the 2 o’clock position to separate the core (i.e., striatum and overlying cortex) from the penumbra (adjacent cortex). Thereafter, tissues were homogenized in lysis buffer containing protease inhibitors (10 mM β-mercaptoethanol, 1 mM phenylmethyl sulfonyl fluoride, and 1 mM sodium orthovanadate) and centrifuged at 10,000 rpm for 10 min at 4℃. Supernatants were collected and stored at −40℃.

### Coimmunoprecipitation of EGFR and SOS1, and Raf-1 and Phospho-Akt

The brains were homogenized with EBC buffer (50 mM Tris-HCl, pH 8.0, 120 mM NaCl, 0.5% Nonidet P-40, 100 mM NaF, 10 mM β-mercaptoethanol, 1 mM phenylmethyl sulfonyl fluoride, and 1 mM sodium orthovanadate) and centrifuged at 10,000 rpm for 10 min at 4℃. Supernatants were collected and stored at −40℃. The coimmunoprecipitation was performed with anti-EGFR antibody or with anti-Raf-1 antibody and protein agarose bead. The nitrocellulose membranes were incubated with the anti-SOS1 antibody or anti-EGFR antibody at 1 × 500 or at 1 × 800 dilution, or with the anti-phospho-Akt (p-AKT) antibody or anti-AKT antibody at 1 × 500 or at 1 × 1,000 dilution for 2 hr at 25℃.

### Western Blotting for Phosphorylated ERK and p-AKT, p-Raf Ser259, and p-Raf Ser338

The protein content was determined in the homogenates by the Bradford method (Bradford, 1976), using bovine serum albumin as the standard. Samples 80 μg of protein were applied on the slab gel of 10% polyacrylamide. After transfer to nitrocellulose membranes, the samples were blocked by 5% skimmed milk powder in TBS-T (30 mM Tris-HCl, 125 mM NaCl, 0.1% Tween 20) for 1 hr. The nitrocellulose membranes were incubated with the first antibody, specific to phosphorylated ERK (p-ERK_1/2_) at 1 × 1,000 dilution, ERK_1/2_ at 1 × 3,000 dilution, p-AKT at 1 × 1,000 dilution, AKT at 1 × 1,000 dilution, p-Raf Ser259 at 1 × 1,000 dilution, p-Raf Ser338 at 1 × 1,000 dilution, and Raf-1 at 1 × 1,000 dilution for 2 hr at room temperature. After washing, specific binding was detected by goat-anti-mouse or goat-anti-rabbit horseradish peroxidase-conjugated secondary antibody at 1 × 1,500 dilution or at 1 × 3,000 dilution. Staining was visualized by ECL detection reagents. Digital images obtained using Gel-Imaging System (Tanon 4200, Shanghai, China). Optical density for each band was assessed using the Window Alpha-Ease TM FC 32-bit software.

### Chemicals

LY294002 hydrochloride, TCN, and catalase were purchased from Sigma (St. Louis, MO, USA). AG1478, GM6001 (N-[(2 R)-2-(hydroxamidocarbonylmethyl)-4-methylpentanoyl]-L-tryptophan methylamide), and U0126 were purchased from Calbiochem (Merck, Darmstadt, Germany). Rapamycin was purchased from Cell Signaling Technology (Danvers, MA, USA). BPV was purchased from Santa Cruz Biotechnology (Santa Cruz, CA, USA). Pentobarbital sodium (SCRC 69020180) was obtained from Shanghai Chemical Reagent Co., Ltd (Shanghai, China). Santa Cruz Biotechnology (Santa Cruz, CA, USA) supplied first antibodies, rabbit polyclonal antibody raised against ERK (K-23): sc-94, mouse monoclonal antibody against p-ERK (E-4): sc-7383, rabbit polyclonal antibody against SOS1 (C-23): sc-256, rabbit polyclonal antibody against p-AKT1/2/3 (Ser473): sc-33437, rabbit polyclonal antibody against phosphorylated tyrosine of EGFR (pEGFR) (Tyr 1173-R): sc-12351-R, and mouse monoclonal antibody used for immunoprecipitation of Raf-1(E-10): sc-7267. The sheep polyclonal antibody used for immunoprecipitation of EGFR (06–129), as well as Protein G agarose bead slurry (16–266), was purchased from Upstate Biotechnology (Lake Placid, NY, USA). The rabbit polyclonal antibody against EGFR (2232), rabbit polyclonal antibody against c-Raf (9422), rabbit polyclonal antibody against p-c-Raf (Ser338) (9427), rabbit polyclonal antibody against p-c-Raf (Ser259) (9421), and rabbit polyclonal antibody against Akt (9272) used for western blotting were purchased from Cell Signaling Technology (Danvers, MA, USA). ECL detection reagents were purchased from Amersham Biosciences (Buckinghamshire, UK).

### Statistics

The differences between individual groups were analyzed by one-way analysis of variance followed by Fisher’s least significant difference test. The comparisons were made between the core region on the lesion side with the same region on the contralateral side, and the penumbra on lesion side with the corresponding region on the contralateral side, to identify the significance related to ischemia/reperfusion. The level of significance was set at *p* < .05.

## Results

### EGFR, ERK_1/2_, and AKT Activation During Ischemia

**Figure 1.**
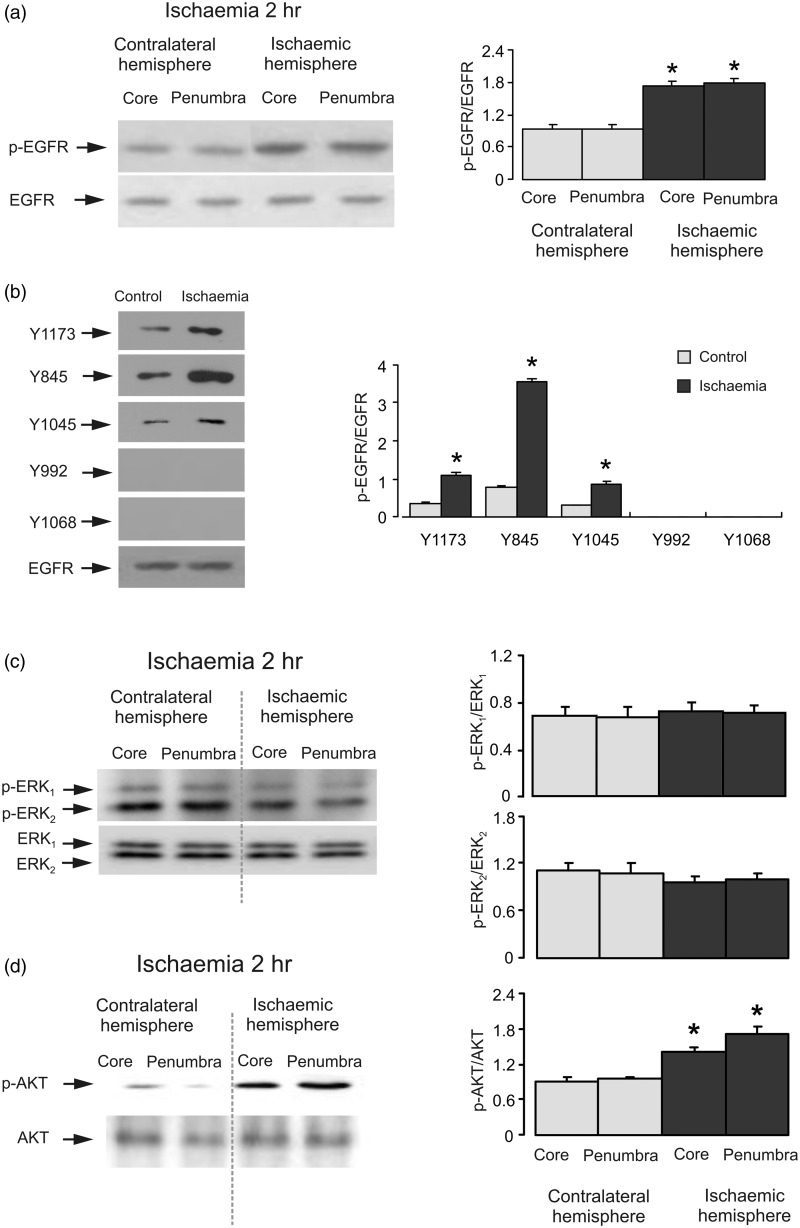
Ischemia induces EGFR and AKT phosphorylation, but not ERK_1/2_ phosphorylation. Two hr of focal ischemia was induced by MCAO. (a) Bands of 170 kDa represent phosphorylated tyrosine of EGFR (pEGFR; upper rows) or total EGFR (lower rows). (b) Bands of 170 kDa represent p-Y1173, p-Y845, p-Y1045, p-Y992 or p-Y1068 (phosphorylated Y1173, Y845, Y1045, Y992, and Y1068 of EGFR; upper rows) or total EGFR (lower rows). Immunoblots from a representative experiment. Similar results are obtained from three independent experiments. Average EGFR phosphorylation is quantified as ratios between pEGFR and EGFR. Standard error of the mean (SEM) values are indicated by vertical bars. *Statistically significant (*p* < .05) difference from contralateral hemisphere. (c) Bands of 44 and 42 kDa represent phosphorylated ERK_1_ (p-ERK_1_) and phosphorylated ERK_2_ (p-ERK_2_), respectively (upper rows), or total ERK_1_ and ERK_2_ (lower rows). (d) Bands of 60 kDa represent phosphorylated AKT (p-AKT; upper rows) or total AKT (lower rows). Immunoblots from a representative experiment. Similar results are obtained from three independent experiments. Average ERK_1/2_ phosphorylation is quantified as ratios between p-ERK_1/2_ and ERK_1/2_. Average AKT phosphorylation is quantified as ratios between p-AKT and AKT. SEM values are indicated by vertical bars. *Statistically significant (*p* < .05) difference from the corresponding core or penumbra from contralateral hemisphere, but not from each other. EGFR = epidermal growth factor receptor; pEGFR = phosphorylated tyrosine of EGFR; ERK = extracellular signal-regulated kinase; p-ERK = phosphorylated ERK; AKT = protein kinase B; p-AKT = phospho-Akt; MCAO = middle cerebral artery occlusion.

First, we investigated EGFR status by quantifying its phosphorylation by immunoprecipitation and specific antibodies. EGFR was precipitated with a specific antibody. Western blot showed an increase of EGFR activity during ischemia that was confirmed by an antibody specific to tyrosine phosphorylation sites in both core and penumbra in ischemic hemisphere (Figure 1(a)). With site-specific antibodies, phosphorylation of Y1173, Y845, and Y1045 was enhanced after 2 hr of ischemia (Figure 1(b)). The greatest stimulation was observed at Y845 that was around 400% of contralateral hemisphere. In contrast, Y992 and Y1068 were not phosphorylated in both contralateral and ischemia hemispheres.

**Figure 2.**
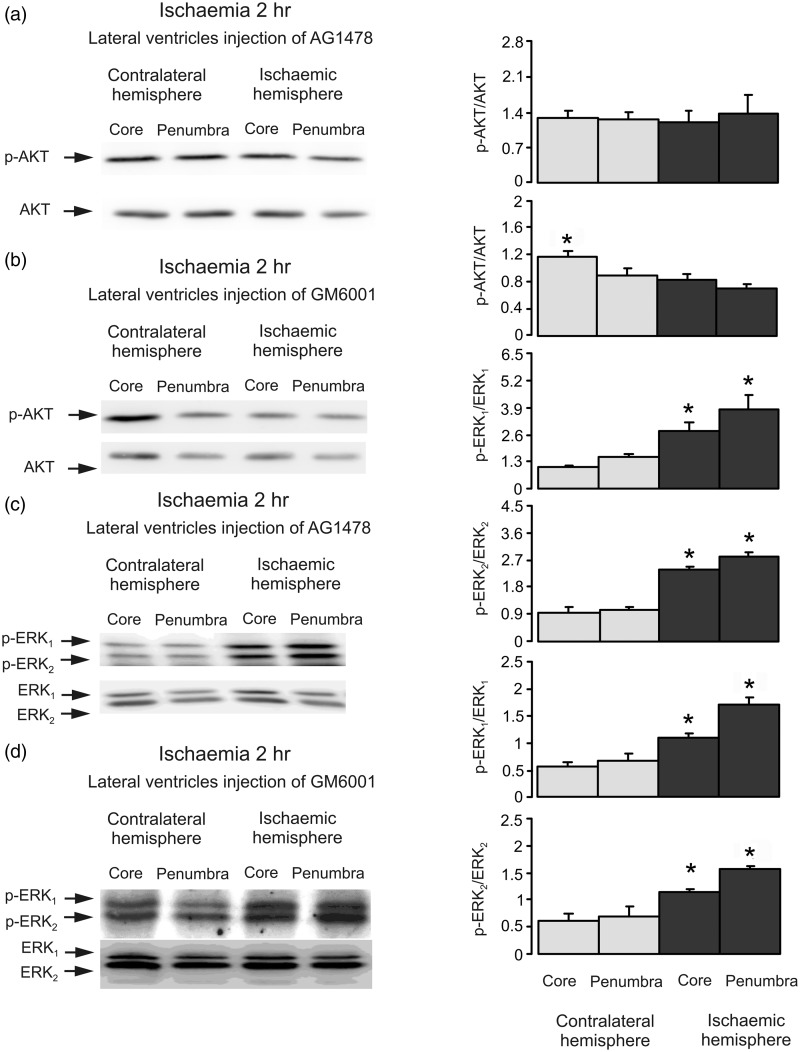
The activity of EGF receptor is required in ischemia-induced AKT phosphorylation. Two hr of focal ischemia was induced by MCAO. AG1478, an inhibitor of EGFR (a and c) or GM6001, an inhibitor of metalloproteinase (b and d) was injected intraventricularly before ischemia. (a and b) Bands of 60 kDa represent p-AKT or total AKT. (c and d) Bands of 44 and 42 kDa represent p-ERK_1_ and p-ERK_2_, or total ERK_1_ and ERK_2_. Immunoblots from a representative experiment. Similar results are obtained from three independent experiments. (a and b) Average AKT phosphorylation is quantified as ratios between p-AKT and AKT. *Statistically significant (*p* < .05) difference from the corresponding core from ischemic hemisphere. (c and d) Average ERK_1/2_ phosphorylation is quantified as ratios between p-ERK_1/2_ and ERK_1/2_. SEM values are indicated by vertical bars. *Statistically significant (*p* < .05) difference from the corresponding core or penumbra from contralateral hemisphere, but not from each other. ERK = extracellular signal-regulated kinase; p-ERK = phosphorylated ERK; AKT = protein kinase B; p-AKT = phospho-Akt; AG1478 = tyrphostin 4-(3-chloroanilino)-6,7-dimethoxyquinazoline; GM6001 = (N-[(2 R)-2-(hydroxamidocarbonylmethyl)-4-methylpentanoyl]-L-tryptophan methylamide); MCAO = middle cerebral artery occlusion.

Next, we analyzed ERK_1/2_ and AKT phosphorylation by Western blot. There was no significant difference in ERK_1/2_ phosphorylation neither in the core nor in the penumbra areas between ischemic and contralateral hemispheres after 2 hr of ischemia (Figure 1(c)). In contrast, AKT phosphorylation was significantly increased in both core and penumbra regions of ischemic hemisphere (Figure 1(d)). To identify the role for EGFR transactivation, the AG1478, an inhibitor of EGFR or GM6001, an inhibitor of Zn^2+^-dependent metalloproteinase and therefore the subsequent shedding of growth factor(s) were injected intraventricularly before ischemia. Both drugs abolished phosphorylation of AKT (Figure 2(a) and (b)), indicating the involvement of EGFR activity and shedding of growth factor(s). In the presence of both AG1478 and GM6001, we observed an increase of ERK_1/2_ phosphorylation, suggesting that ERK_1/2_ phosphorylation during ischemia is suppressed by activation of EGFR and its downstream signals (Figure 2(c) and (d)). Obviously, this ERK_1/2_ phosphorylation is not mediated by EGFR because it is increased but not inhibited in the presence of AG1478 and GM6001.

### SOS1 and Raf-1 Activation During Ischemia

**Figure 3.**
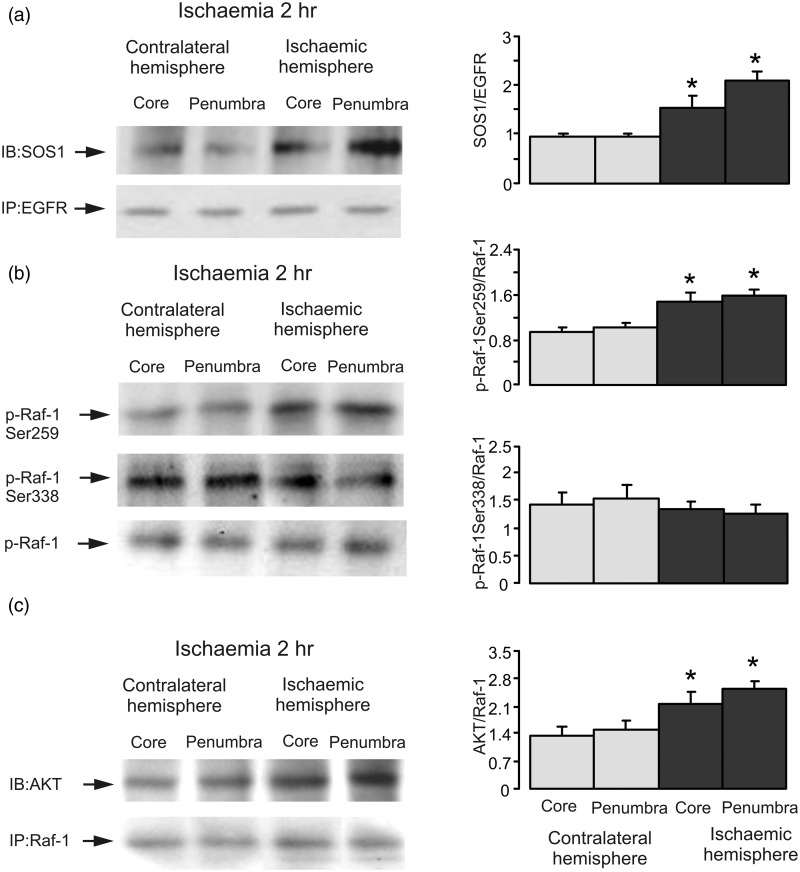
Ischemia increases interactions between SOS1 and EGFR or between p-AKT and Raf-1 and stimulates Raf-1 Ser259 phosphorylation, but not Ser338 phosphorylation. Two hr of focal ischemia was induced by MCAO. (a) Coimmunoprecipitation was performed with EGFR antibody, and blots were stained with either SOS1 antibody or EGFR antibody. Bands of 170 kDa represent SOS1 (upper rows) or EGFR (lower rows), respectively. (b) Bands of 74 kDa represent p-Raf-1 Ser259 (first rows), Ser338 (seconnd rows), or total Raf-1 (bottom rows). (c) Coimmunoprecipitation was performed with Raf-1 antibody, and blots were stained with either p-AKT antibody or Raf-1 antibody. Bands of 60 and 74 kDa represent phosphorylated AKT (p-AKT; upper rows) or Raf-1 (lower rows), respectively. Immunoblots from a representative experiment. Similar results are obtained from three independent experiments. (a) Average binding of SOS1 and EGFR is quantified as ratios between SOS1 and EGFR. (b) Average phosphorylation of Ser259 or Ser338 is quantified as ratios between Ser259 and Raf-1 or between Ser338 and Raf-1. Average binding of p-AKT and Raf-1 is quantified as ratios between p-AKT and Raf-1. SEM values are indicated by vertical bars. *Statistically significant (*p* < .05) difference from the corresponding core or penumbra from contralateral hemisphere, but not from each other. SOS1 = Son of sevenless 1; EGFR = epidermal growth factor receptor; AKT = protein kinase B; MCAO = middle cerebral artery occlusion.

Subsequently, we examined the interactions between SOS1 and EGFR. Coimmunoprecipitation of SOS1 with EGFR antibody showed a significant increase of binding of SOS1 to EGFR after 2-hr ischemia in both core and penumbra areas in ischemic hemisphere as expected (Figure 3(a)), indicating that ischemia has a stimulatory (rather than inhibitory) effect on the interaction and SOS1 activity and that ischemia interferes with ERK_1/2_ phosphorylation at a site downstream of SOS1. Taking in account high levels of AKT activity during ischemia and its inhibitory effect on Raf-1 (Zimmermann and Moelling, 1999; Moelling et al., 2002), we further analyzed the phosphorylation of inhibitory Raf-1 Ser259 site and stimulatory Raf-1 Ser338 site of Raf-1 by Western blot. Two hr of ischemia enhanced Raf-1 Ser259 phosphorylation significantly but had no effect on Raf-1 Ser338 (Figure 3(b)), suggesting that a decrease of Raf-1 activity is likely to be responsible for the ischemia-induced MAPK/ERK_1/2_ inactivation.

### Crosstalk Between PI3K/AKT and Raf-1 During Ischemia

**Figure 4.**
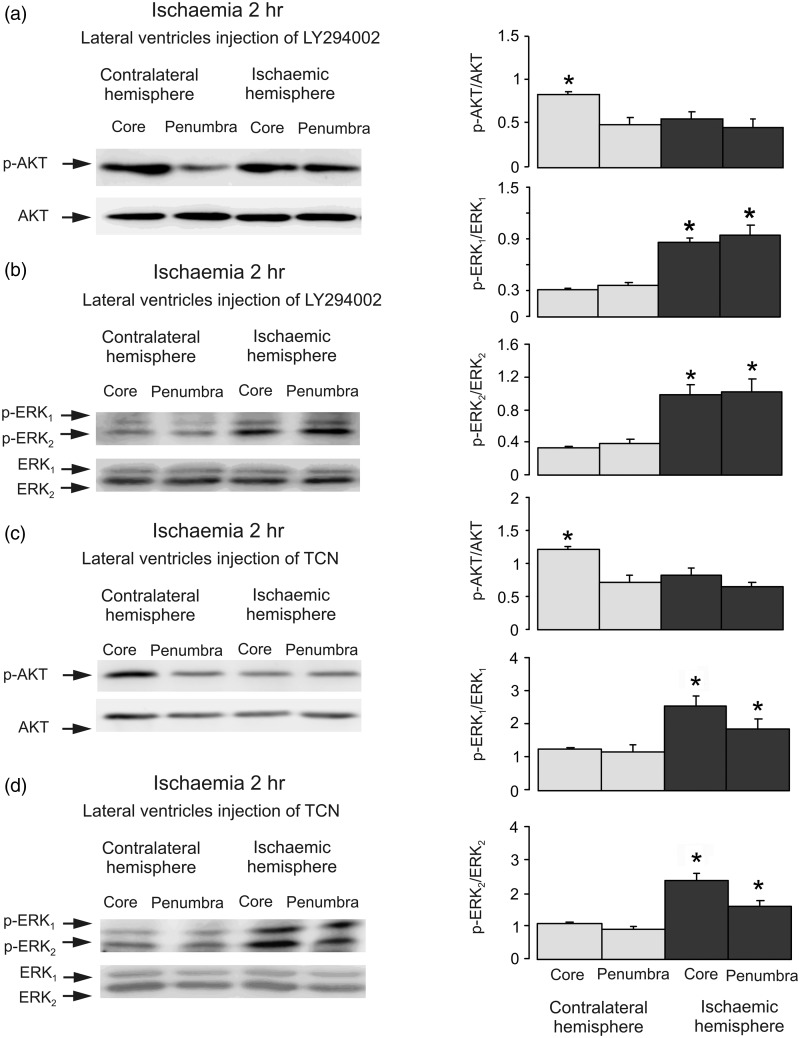
Both PI3K and AKT activities are required in ischemia-induced inhibition of ERK_1/2_ phosphorylation. Two hr of focal ischemia was induced by MCAO. LY294002, an inhibitor of PI3K (a and b) or TCN, an inhibitor of AKT (c and d) was injected intraventricularly before ischemia. (a and c) Bands of 60 kDa represent phosphorylated AKT (p-AKT; upper rows) or total AKT (lower rows). (b and d) Bands of 44 and 42 kDa represent p-ERK_1_ and p-ERK_2_ (upper rows), or total ERK_1_ and ERK_2_ (lower rows). Immunoblots from a representative experiment. Similar results are obtained from five independent experiments. (a and c) Average AKT phosphorylation is quantified as ratios between p-AKT and AKT. SEM values are indicated by vertical bars. *Statistically significant (*p* < .05) difference from the corresponding core from contralateral hemisphere. (b and d) Average ERK_1/2_ phosphorylation is quantified as ratios between p-ERK_1/2_ and ERK_1/2_. SEM values are indicated by vertical bars. *Statistically significant (*p* < .05) difference from other groups, but not from each other. LY294002 = 2-(4-morpholinyl)-8-phenyl-1(4H)-benzopyran-4-one hydrochloride; TCN = triciribine hydrate; ERK = extracellular signal-regulated kinase; p-ERK = phosphorylated ERK; AKT = protein kinase B; p-AKT = phospho-Akt; MCAO = middle cerebral artery occlusion.

To monitor interactions between p-AKT and Raf-1, we performed coimmunoprecipitation of p-AKT with a specific antibody to Raf-1. Western blot shows that binding of p-AKT to Raf-1 was stimulated by ischemia (Figure 3(c)). Intraventricular injection of LY294002, an inhibitor of PI3K, performed prior to ischemic insult abolished the stimulation of AKT phosphorylation (Figure 4(a)) but increased ERK_1/2_ phosphorylation, suggesting a recovery of ERK_1/2_ phosphorylation by inhibition of PI3K (Figure 4(b)), and indicating the inhibitory effect of PI3K/AKT on Raf-1/MAPK/ERK_1/2_ pathway. AKT inhibitor, TCN also inhibited AKT phosphorylation (Figure 4(c)) and induced an increase of ERK_1/2_ phosphorylation in both core and penumbra areas in ischemic hemisphere as expected (Figure 4(d)), suggesting that Raf-1 Ser259 is directly phosphorylated by AKT.

### EGFR, ERK_1/2_, and AKT Activation During Reperfusion

**Figure 5.**
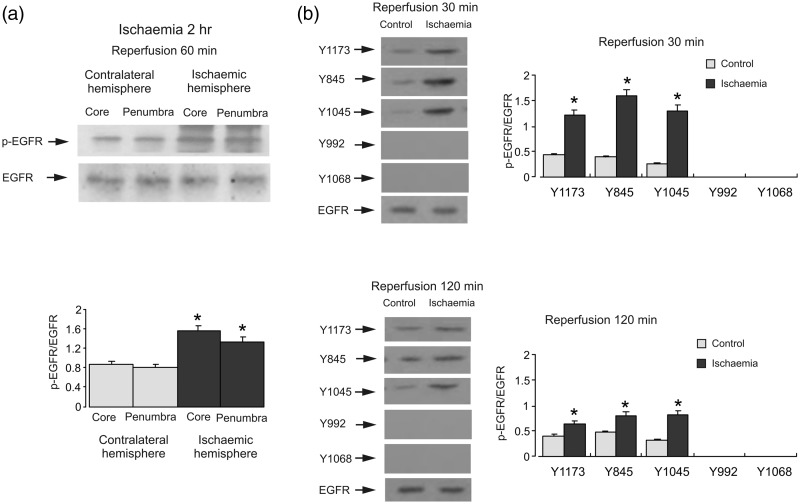
EGFR phosphorylation during reperfusion. Two hr of focal ischemia was followed by 1- (a), 0.5- or 2-hr (b) reperfusion. (a) Bands of 170 kDa represent phosphorylated tyrosine of EGFR (pEGFR; upper rows) or total EGFR (lower rows). (b) Bands of 170 kDa represent pY1173, pY845, pY1045, pY992, or pY1068 (phosphorylated Y1173, Y845, Y1045, Y992, and Y1068 of EGFR; upper rows) or total EGFR (lower rows). Immunoblots from a representative experiment. Similar results are obtained from three independent experiments. Average EGFR phosphorylation is quantified as ratios between pEGFR and EGFR. SEM values are indicated by vertical bars. *Statistically significant (*p* < .05) difference from contralateral hemisphere. EGFR = epidermal growth factor receptor; pEGFR = phosphorylated tyrosine of EGFR.

The increase of EGFR activity after reperfusion was confirmed by an antibody specific to tyrosine phosphorylation sites of EGFR (Figure 5(a)). With site-specific antibodies, phosphorylation of Y1173, Y845, and Y1045 was significantly increased to 281.0 ± 3.7%, 410.5 ± 6.0%, and 508.0 ± 3.3% (*n* = 3) of contralateral hemisphere, respectively, after 0.5-hr reperfusion (Figure 5(b)). The levels of phosphorylation at all three sites were declined after 2-hr reperfusion. Again, Y992 and Y1068 were not phosphorylated in both contralateral and ischemic hemispheres.

**Figure 6.**
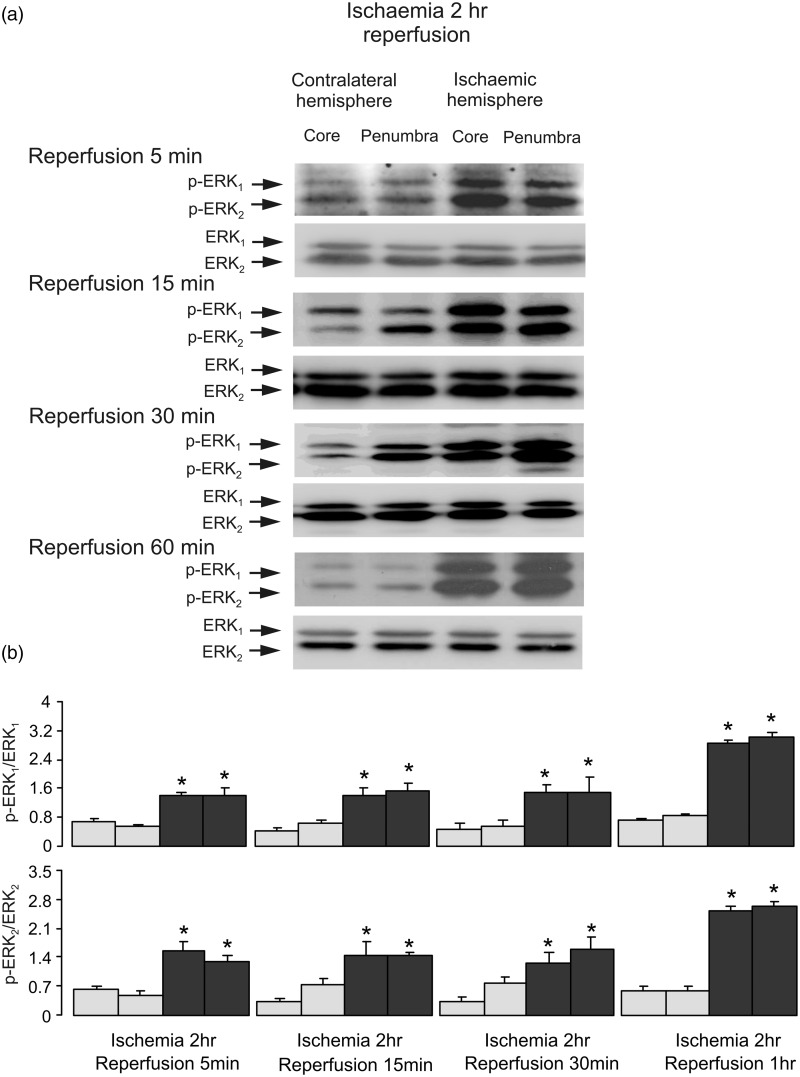
ERK_1/2_ phosphorylation during reperfusion. Two hr of focal ischemia was followed by 5-, 15-, 30-min and 1-hr reperfusion. Bands of 44 and 42 kDa represent p-ERK_1_ and p-ERK_2_ (upper rows), or total ERK_1_ and ERK_2_ (lower rows). (a) Immunoblots from a representative experiment. Similar results are obtained from three independent experiments. Average ERK_1/2_ phosphorylation is quantified as ratios between p-ERK_1/2_ and ERK_1/2_ (b). SEM values are indicated by vertical bars. *Statistically significant (*p* < .05) difference from the corresponding core or penumbra from contralateral hemisphere, but not from each other. ERK = extracellular signal-regulated kinase; p-ERK = phosphorylated ERK.

**Figure 7.**
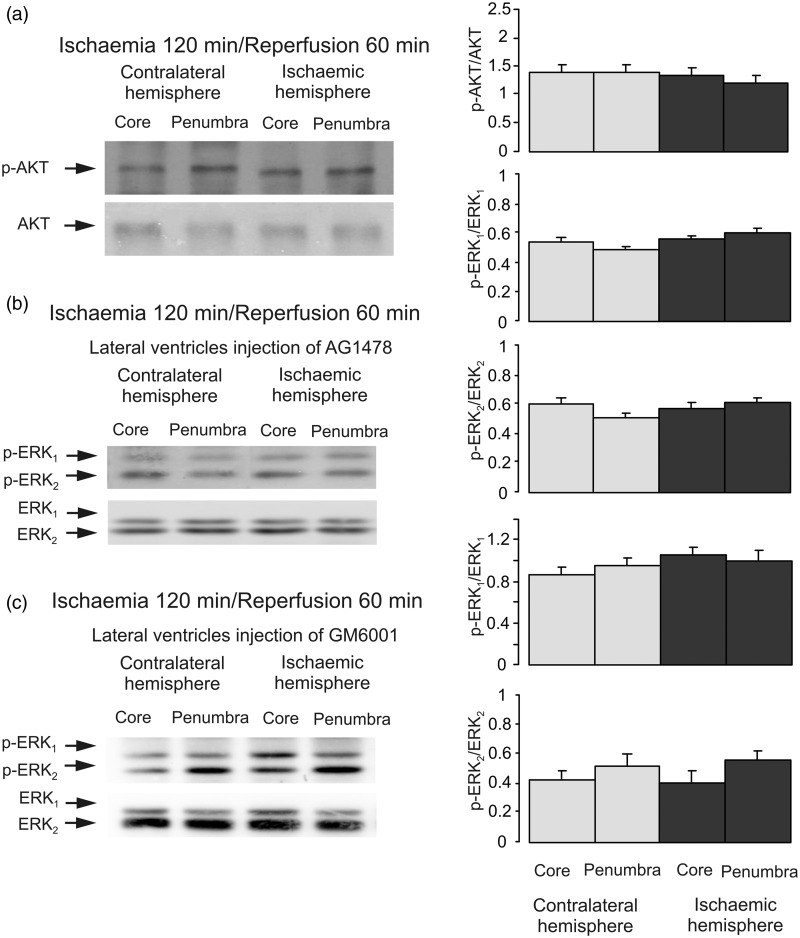
AKT and EGFR activation during reperfusion. Two hr of focal ischemia was followed by 1-hr reperfusion (a). AG1478, an inhibitor of EGFR (b) or GM6001, an inhibitor of metalloproteinase (c) was injected intraventricularly after 2-hr ischemia but before 1-hr reperfusion. (a) Bands of 60 kDa represent p-AKT or total AKT. (b and c) Bands of 44 and 42 kDa represent p-ERK_1_ and p-ERK_2_, or total ERK_1_ and ERK_2_. Immunoblots from a representative experiment. Similar results are obtained from four independent experiments. (a) Average AKT phosphorylation is quantified as ratios between p-AKT and AKT. (b and c) Average ERK_1/2_ phosphorylation is quantified as ratios between p-ERK_1/2_ and ERK_1/2_. SEM values are indicated by vertical bars. ERK = extracellular signal-regulated kinase; p-ERK = phosphorylated ERK; AKT = protein kinase B; p-AKT = phospho-Akt; AG1478 = tyrphostin 4-(3-chloroanilino)-6,7-dimethoxyquinazoline; GM6001 = (N-[(2 R)-2-(hydroxamidocarbonylmethyl)-4-methylpentanoyl]-L-tryptophan methylamide).

To analyze ERK_1/2_ phosphorylation during reperfusion, we performed experiments with 2-hr ischemia followed by 5-, 15-, 30-min, and 1-hr reperfusion. Phosphorylation of ERK_1/2_ was significantly increased at all the time points (Figure 6), suggesting that ERK_1/2_ phosphorylation is induced by the reperfusion as such, no matter the duration. In contrast, there is no significant difference in AKT phosphorylation in both core and penumbra areas between ischemic and contralateral hemispheres, indicating that the increase of AKT phosphorylation by ischemia returns to control level after reperfusion (Figure 7(a)).

The stimulation of ERK_1/2_ phosphorylation during reperfusion was significantly decreased by injection of AG1478, an inhibitor of EGFR (Figure 7(b)) and by GM6001, an inhibitor of metalloproteinase at the end of ischemia but before reperfusion (Figure 7(c)), indicating the involvement of EGFR activation.

### Crosstalk Between PI3K/AKT and Raf/MAPK/ERK_1/2_ During Reperfusion

**Figure 8.**
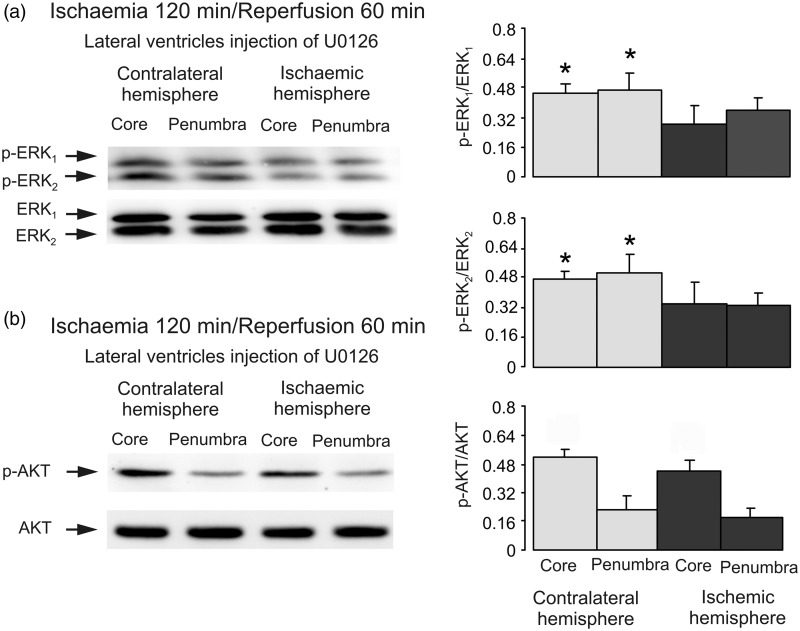
MAPK/ERK_1/2_ has no effect on AKT phosphorylation during reperfusion. Two hr of focal ischemia was followed by 1-hr reperfusion. U0126, an inhibitor of MAPK (a and b) was injected intraventricularly after 2-hr ischemia but before 1-hr reperfusion. (a) Bands of 44 and 42 kDa represent p-ERK_1_ and p-ERK_2_, or total ERK_1_ and ERK_2_. (b) Bands of 60 kDa represent p-AKT or total AKT. Immunoblots from a representative experiment. Similar results are obtained from three independent experiments. (a) Average ERK_1/2_ phosphorylation is quantified as ratios between p-ERK_1/2_ and ERK_1/2_. SEM values are indicated by vertical bars. *Statistically significant (*p* < .05) difference from other groups, but not from each other. (b) Average AKT phosphorylation is quantified as ratios between p-AKT and AKT. SEM values are indicated by vertical bars. ERK = extracellular signal-regulated kinase; p-ERK = phosphorylated ERK; AKT = protein kinase B; p-AKT = phospho-Akt; U0126 = 1,4-diamino-2,3-dicyano-1,4-bis[2-aminophenylthio] butadiene.

**Figure 9.**
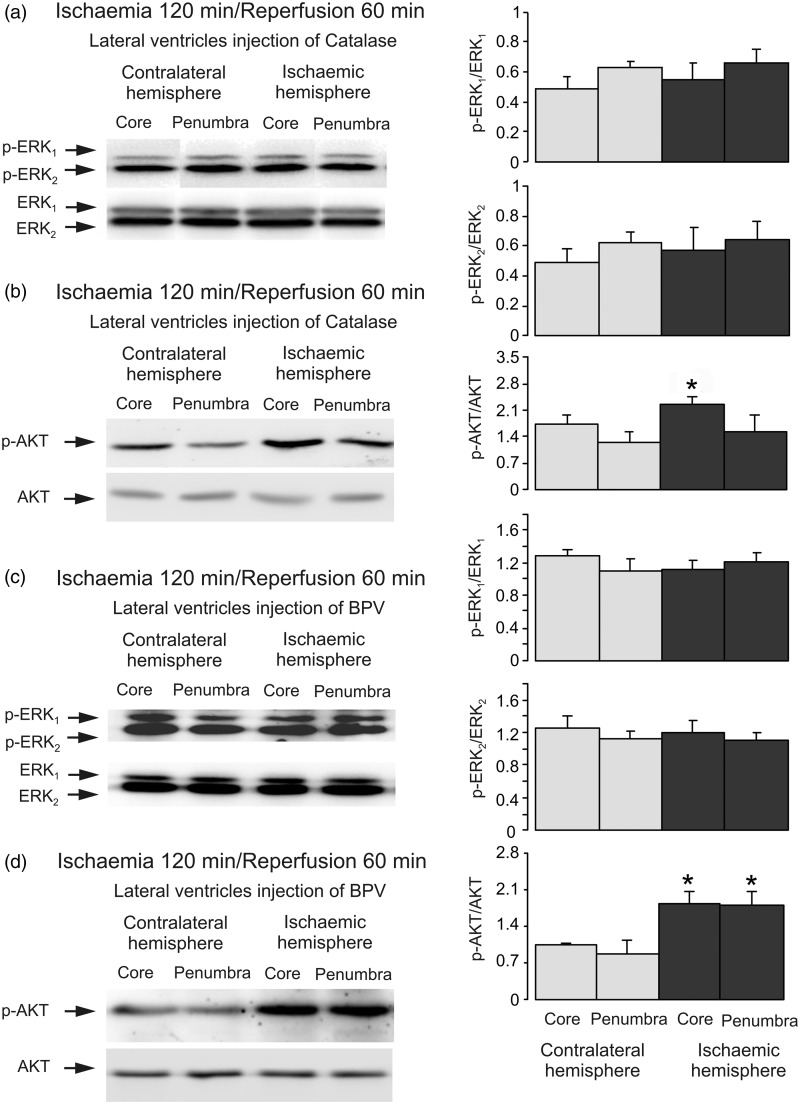
ROS and PTEN are involved in AKT inhibition during reperfusion. Two hr of focal ischemia was followed by 1-hr reperfusion. Catalase, an inhibitor of ROS (a and b) or BPV, an inhibitor of PTEN (c and d) was injected intraventricularly after 2-hr ischemia but before 1-hr reperfusion. (a and c) Bands of 44 and 42 kDa represent p-ERK_1_ and p-ERK_2_, or total ERK_1_ and ERK_2_. (b and d) Bands of 60 kDa represent p-AKT or total AKT. Immunoblots from a representative experiment. Similar results are obtained from four independent experiments. (a and c) Average ERK_1/2_ phosphorylation is quantified as ratios between p-ERK_1/2_ and ERK_1/2_. (b and d) Average AKT phosphorylation is quantified as ratios between p-AKT and AKT. SEM values are indicated by vertical bars. *Statistically significant (*p* < .05) difference from the corresponding core or penumbra from contralateral hemisphere, but not from each other. ERK = extracellular signal-regulated kinase; p-ERK = phosphorylated ERK; AKT = protein kinase B; p-AKT = phospho-Akt; BPV = bisperoxovanadium.

Intracerebroventricular injection of U0126, an inhibitor of MAPK after 2 hr of ischemia but before 1-hr reperfusion reversed the stimulation of p-ERK. Both p-ERK_1_ and p-ERK_2_ are significantly lower in the core and penumbra on lesion side than on the contralateral side as expected, while no effect on AKT phosphorylation was observed (Figure 8(a) and (b)), indicating that MAPK/ERK_1/2_ has no inhibitory effect on PI3K/AKT signal pathway during reperfusion. In Figure 8(b), p-AKT is significantly higher in the core than penumbra in both contralateral and ischemic sides. However, there is no difference between the cores in contralateral and ischemic sides, suggesting the high p-AKT in core is not related to ischemia. Finally, we injected catalase, an enzyme degrading hydrogen peroxide and hence counteraction effects of ROS after 2-hr ischemia but before 1-hr reperfusion. Injection of catalase abolished stimulation of ERK_1/2_ phosphorylation (Figure 9(a)), and restored AKT phosphorylation (Figure 9(b)). BPV, an inhibitor of phosphatase and tensin homolog (PTEN) had similar effect (Figure 9(c) and (d)).

## Discussion

Experiments on myocardial ischemia/reperfusion model demonstrated that ERK phosphorylation depends on reperfusion (Omura et al., 1999), but the underlying mechanism remains unknown. Even less is known about the effects of ischemia/reperfusion in the brain. In the present article, we performed systematic analysis of EGFR and its two signal pathways Raf/MAPK/ERK_1/2_ and PI3K/AKT during ischemia and reperfusion using rat MCAO model. We have investigated (a) the EGFR transactivation and the activations of PI3K/AKT and Raf/MAPK/ERK_1/2_ signal pathways during ischemia, (b) the interaction between PI3K/AKT and Raf/MAPK/ERK_1/2_ signal pathways during ischemia, (c) the EGFR transactivation and the activations of PI3K/AKT and Raf/MAPK/ERK_1/2_ signal pathways during reperfusion, and (d) the interaction of PI3K/AKT and Raf/MAPK/ERK_1/2_ signal pathways during reperfusion.

First, we found that there was no increase of ERK_1/2_ phosphorylation during ischemia, but phosphorylation significantly increased in reperfusion. This is similar to myocardial ischemia/reperfusion model (Omura et al., 1999). Phosphorylation of AKT was significantly enhanced during ischemia, and it was inhibited by both AG 1478, an inhibitor of EGFR and GM6001, an inhibitor of Zn^2+^-dependent metalloproteinase, indicating an involvement of EGFR transactivation. Activation of EGFR was further confirmed by EGFR phosphorylation at Y1173, Y845, and Y1045 cites during ischemia. In some cases (Figures 2, 5, and 9), the p-AKT in the core is higher than in the penumbra in the contralateral side. We do not know the physiological significance of this phenomenon yet, but it seems to be unrelated to ischemia or reperfusion. The EGF can induce phosphorylation of all five tyrosine phosphorylation sites of EGFR (Peng et al., 2010). Among these sites, Y992, Y1173, and Y1045 are autophosphorylation sites, with Y1173 as a major one and Y992 as a minor one. The Y845 is known to be a major Src phosphorylation site (Biscardi et al., 1999; Tice et al., 1999; Wu et al., 2002). Ultraviolet B radiation was also shown to promote the phosphorylation of multiple tyrosine residues of EGFR, including Y845, and subsequently potentiate PI3K/AKT phosphorylation and inactivation of ERK_1/2_ signaling cascade in epidermal keratinocytes (Iordanov et al., 2002); this response being cell-type-specific. Y1068 is not phosphorylated in the brain *in vivo* (Du et al., 2009) neither it is phosphorylated in cultured astrocytes, unless the latter were stimulated by EGF (Peng et al., 2010) or by ammonium (Dai et al., 2013).

Second, we found that despite the absence of ERK_1/2_ phosphorylation, the interaction between EGFR and SOS1 was seemingly normal and was stimulated by ischemia. However, the stimulatory phosphorylation site of Raf-1, Ser338 was not phosphorylated by ischemia. Instead, the phosphorylation of inhibitory site Ser259 was enhanced. This indicates that the Raf/MAPK/ERK_1/2_ signal pathway is inhibited at the site of Raf-1. Phosphorylation of Ser338 is required for Raf activation and also potentiates the association between Raf and MAPK (Chaudhary et al., 2000; Xiang et al., 2002), whereas phosphorylation of Raf-1 Ser259 interfered with membrane association and activity of Raf-1 (Kubicek et al., 2002). Furthermore, we found that coimmunoprecipitation of AKT with Raf-1 antibody indicated increased interaction between p-AKT and Raf-1 during ischemia. The inhibitory effect of PI3K/AKT on Raf/MAPK/ERK_1/2_ pathway was further confirmed by the increase of ERK phosphorylation by LY294002, an inhibitor of PI3K and triciribine hydrate (TCN), an inhibitor of AKT. All these suggest an inhibitory effect of PI3K/AKT on Raf/MAPK/ERK_1/2_ pathway during ischemic period. Similar inhibition has been reported for other tissues and cells, such as AKT-induced phosphorylation of Raf-1 at Ser259 by insulin-like growth factor 1 in MCF-7, a human breast cancer cell line (Zimmermann and Moelling, 1999; Moelling et al., 2002) and by platelet-derived growth factor in vascular smooth muscle cells (Reusch et al., 2001). Nevertheless, AKT inhibited the ERK_1/2_ phosphorylation in human myotubes (Rommel et al., 1999), that effect possibly not being mediated by the direct phosphorylation of Raf-1 at Ser259 (Garcia et al., 2009).

Third, we found no increase of AKT phosphorylation in reperfusion period. However, ERK phosphorylation was significantly enhanced during reperfusion, and it was inhibited by both AG 1478, an inhibitor of EGFR and GM6001, an inhibitor of Zn^2+^-dependent metalloproteinase, indicating an involvement of EGFR transactivation. The sites of EGFR phosphorylation were the same as ischemic period. As has been reported previously, permanent increase of EGFR and ERK activity during reperfusion and inhibition of EGFR and ERK activity have protective effect (Martin and Pognonec, 2010; Yang et al., 2011).

Fourth, we investigated whether PI3K/AKT pathway is inhibited by Raf/MAPK/ERK_1/2_ pathway during reperfusion. Our data indicate that it is not the case because inhibition of MAPK and ERK_1/2_ does not reinstate AKT phosphorylation. However, inhibition of catalase activity not only abolishes ERK phosphorylation but also restores AKT phosphorylation. This indicates that both ERK_1/2_ stimulation and AKT inhibition during reperfusion are mediated by ROS. ROS-induced EGFR transactivation has been repeatedly reported (Frank and Eguchi, 2003; Papaiahgari et al., 2006; Wolf-Goldberg et al., 2013; Xu et al., 2013). More interesting, ROS stimulates PTEN, a negative regulator of PI3K/AKT signaling (Hlobilková et al., 2003) during reperfusion that in turn releases Raf-MAPK/ERK from inhibitory effect of AKT. This finding is in agreement with the findings that PTEN inhibitors protect cells from ischemia/reperfusion-induced cell damage (Mocanu and Yellon, 2007).

Activity of AKT has been studied in the context of preconditioning in combination with MCAO model (Shibata et al., 2002; Meller et al., 2005). Preconditioning prolonged the AKT phosphorylation and protected neurons in the penumbra in MCAO model (Nakajima et al., 2004). In gerbil transient global ischemia, preconditioning was performed by 2-min sublethal ischemia 3 days before 5-min lethal ischemia. Although AKT played a protective role, the activation of AKT would not occur until 1 day after preconditioning and 12 hr after preconditioning combined with ischemia in CA1 region of hippocampus (Yano et al., 2001). However, in another study, 3.5 min of ischemia induced increase of AKT phosphorylation transiently in 10 to 60 min after reperfusion (Namura et al., 2000). These findings suggest that (a) there is a transient increase of AKT activity after preconditioning, at the early stage of ischemia (1–2 hr), and after several hrs of reperfusion; (b) increase of AKT activity may have protective effects during ischemia and reperfusion periods; and (c) preconditioning-induced AKT phosphorylation may be not related to the protective effect in lethal ischemia, that occurs several days later.

**Figure 10.**
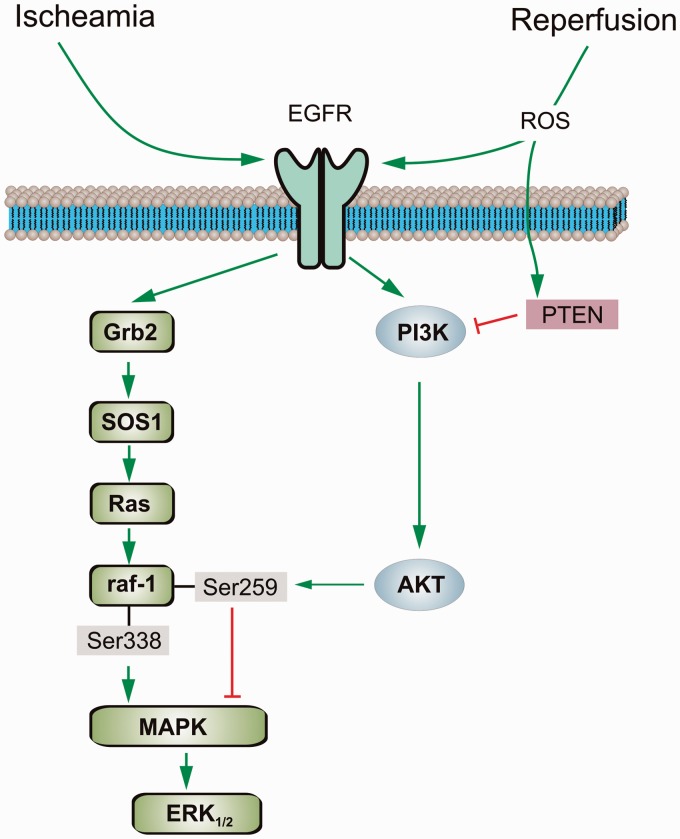
Diagram of crosstalk between Raf/MAPK/ERK and PI3K/AKT signal pathways during brain ischemia and reperfusion. Ischemia induces EGFR transactivation and phosphorylation at Y1173, Y845, and Y1045. The activation of EGFR, in turn, significantly stimulates PI3K/AKT signal pathway. Subsequently, AKT phosphorylates Raf-1 at its inhibitory phosphorylation site Ser259 and inhibits Raf-1 activity. The inhibition of Raf-1 leads to inactivation of its downstream signal MAPK/ERK. During reperfusion, ROS stimulates both EGFR and PTEN. The latter, in turn, inhibits PI3K/AKT signal pathway and restores the activity of Raf-1/MAPK/ERK_1/2_ signal pathway, which is responsible for reperfusion-induced cell damage. EGFR = epidermal growth factor receptor; ROS = reactive oxygen species; PTEN = phosphatase and tensin homolog; SOS1 = Son of sevenless 1; AKT = protein kinase B; MAPK = mitogen-activated protein kinase; ERK = extracellular signal-regulated kinase.

In summary, negative crosstalk exists between Raf/MAPK/ERK_1/2_ and PI3K/AKT signal pathways during brain ischemia/reperfusion. Our study provides the information about the underlying mechanisms of this interaction, that is, during ischemia, AKT phosphorylates Raf-1 at its inhibitory phosphorylation site and decreases Raf/MAPK/ERK_1/2_ activity; during reperfusion, ROS stimulates PTEN that erases the inhibition of AKT on Raf/MAPK/ERK_1/2_ signaling (Figure 10). PI3K/AKT signaling pathway contributes to cell protection in different cells, tissues, and animal models, including focal ischemia and ischemic postconditioning (see Zhao et al., 2013, and references thereby). Nevertheless, the function of Raf/MAPK/ERK_1/2_ signaling may depend on the type of the cells, the time and extent of ERK activation, and the kind of stimulation/insult. ERK phosphorylation mediated by preconditioning (Kovalska et al., 2014) and by numbers of neuroprotective compounds, such as dexmedetomidine, an α_2_-adrenergic agonist, (Du et al., 2009; Zhu et al., 2013) reduces cell damage in brain ischemia. However, inhibition of ERK phosphorylation by administration of MAPK inhibitor prior to ischemia (in both global brain ischemia and MCAO model) decreased cell injury, infarct volume, and neurological deficit (Kindy, 1993; Wang et al., 2003; Castañeda et al., 2009). Therefore, the understanding of crosstalk between Raf/MAPK/ERK_1/2_ and PI3K/AKT signal pathways during brain ischemia/reperfusion may eventually lead to the development of new therapeutic strategy for the disease. Our findings suggest that ROS removes inhibition of AKT on ERK activity during reperfusion. The strong stimulation of ERK activity plays a key role in reperfusion-induced cell damage. Inhibitors of ROS, PTEN, EGFR, and ERK may have therapeutic potential in clinic for brain ischemia.
